# Single Step Laser Transfer and Laser Curing of Ag NanoWires: A Digital Process for the Fabrication of Flexible and Transparent Microelectrodes

**DOI:** 10.3390/ma11061036

**Published:** 2018-06-19

**Authors:** Filimon Zacharatos, Panagiotis Karvounis, Ioannis Theodorakos, Antonios Hatziapostolou, Ioanna Zergioti

**Affiliations:** 1 Physics Department, Zografou Campus, National Technical University of Athens, Athens 15780, Greece; jtheod@mail.ntua.gr (I.T.); zergioti@central.ntua.gr (I.Z.); 2 Campus 1, School of Engineering, University of West Attica, Aigaleo 12243, Greece; panos.karvounis12@gmail.com (P.K.); ahatzi@teiath.gr (A.H.)

**Keywords:** laser curing, laser transfer, silver nanowires, flexible transparent conductive electrodes, heat affected zone, selective heating

## Abstract

Ag nanowire (NW) networks have exquisite optical and electrical properties which make them ideal candidate materials for flexible transparent conductive electrodes. Despite the compatibility of Ag NW networks with laser processing, few demonstrations of laser fabricated Ag NW based components currently exist. In this work, we report on a novel single step laser transferring and laser curing process of micrometer sized pixels of Ag NW networks on flexible substrates. This process relies on the selective laser heating of the Ag NWs induced by the laser pulse energy and the subsequent localized melting of the polymeric substrate. We demonstrate that a single laser pulse can induce both transfer and curing of the Ag NW network. The feasibility of the process is confirmed experimentally and validated by Finite Element Analysis simulations, which indicate that selective heating is carried out within a submicron-sized heat affected zone. The resulting structures can be utilized as fully functional flexible transparent electrodes with figures of merit even higher than 100. Low sheet resistance (<50 Ohm/sq) and high visible light transparency (>90%) make the reported process highly desirable for a variety of applications, including selective heating or annealing of nanocomposite materials and laser processing of nanostructured materials on a large variety of optically transparent substrates, such as Polydimethylsiloxane (PDMS).

## 1. Introduction

Ag Nanowires (NWs) are attracting increasing interest owing to their exquisite properties (electrical conductivity, high optical transparency, high mechanical flexibility), which make them ideal candidates for a variety of components and systems, among which flexible and transparent electrodes for applications in electronics and sensors [1]. For these particular applications, they hold substantial advantages when compared to other nanostructured materials which relate to their low-cost production process and their competitive figures of merit. Due to their high aspect ratio (length/diameter ratio), and the very high conductivity of silver (the most conductive metal), the nanowires can be used in the form of random networks as transparent conductive electrodes with typical Rs < 50 Ohm/sq and T > 85% [1]. Above the percolation threshold, this network is able to transport electrons, while maintaining high transparency through the spaces existing between the nanowires. Their widespread use has contributed to the development of numerous emerging technological applications, e.g., OLEDs [2], solar cells [3,4], transparent film heaters [5,6], flexible touch screens and sensors [7,8,9]. Moreover, single crystalline Ag NWs are usually grown using low-cost solution based processes [10] adding further value to this nano-material in terms of cost effectiveness. Although random NW networks can be fabricated with simple coating techniques [4,11], achieving control and accuracy over the geometry and lateral dimension of the network is technologically attractive for applications in flexible electronics and sensors. Numerous previous articles have highlighted a variety of microfabrication and patterning techniques which are compatible with Ag NWs processing, such as chemical etching [12], laser ablation [13,14] or inkjet printing [15]. Etching or ablation methods are subtractive and can easily induce damage to underlying sensitive materials, whereas inkjet printing, although mask-less and contact-less, is compatible only with a very restricted range of material viscosities (inks in the order to 1–100 cP). Furthermore, a large number of published reports highlight the beneficial effect of thermal or light induced annealing of Ag NW networks in the improvement of their electrical properties. In particular, oven [16,17], laser [18] and even sunlight [19] annealing have evidently ameliorated the performance of photovoltaics and powering of electronic devices. At the same time, Laser Direct Writing, which encompasses Laser Induced Transfer, Laser Sintering and Laser Patterning, of functional materials has emerged as a reliable, micrometer scale resolution, versatile fabrication tool for flexible electronics. However, a limited number of reports referring to Laser Induced Forward Transfer (LIFT), laser patterning and laser processing of Ag NWs in general can be found. In Reference [20], Araki et al. have developed highly conductive electrodes (Rs < 100 Ohm/sq) with 80% total light transparency, by LIFT of pixels comprising Ag NWs incorporated in a resin binder matrix using a Triazene Polymer as a sacrificial layer. Moreover, laser patterning [21] and laser sintering (annealing) [14] of Ag NW networks has been reported. Although subtractive laser processes (i.e., laser ablation and patterning) have been demonstrated for the fabrication of transparent conductive electrodes comprising Ag NWs, the reported process stands out due to the following significant advantages: It is a digital additive process, therefore it can benefit from all of the key points an additive process has to offer, i.e., it can selectively transfer pixels on a predesignated location with submicron accuracy, even on top of pre-patterned samples or pre-existing stacks of layers. Moreover, the amount of donor material that is utilized corresponds to the exact amount of material required for the application. On the contrary, subtractive laser processing would require excessive material to be removed for the patterning of the desired electrode and would possibly compromise the quality of any preexisting structures underneath or around the area of interest. In effect, the laser induced transfer of solid pixels with complex geometries [22], nanopatterned structures [23] and even multi-stacked layers with opto-electronic functionality [24], have been demonstrated over the past decade, highlighting the versatility of the laser induced transfer technology for the digital fabrication of structures and devices with resolutions down to 1 μm. In addition, the authors in previous reports have demonstrated the employment of Laser Direct Writing for the digital fabrication of highly conductive microelectrodes [25] and low-loss Radio Frequency (RF) transmission lines with resolutions down to 1 μm [26], using Ag nanoparticle ink dispersions. Other papers of the reporting group have clearly shown the applicability of the LIFT process for the direct transfer of intact solid pixels of organic semiconducting materials [27,28] and flakes of 2D layered materials [29]. In this work, we demonstrate a novel single-step laser transfer and laser curing approach of Ag NWs pixels and patterns for the fabrication of flexible and transparent conductive micro-electrodes. Typically the nanowires are 1–20 µm long with diameters of 40–80 nm. The nanowires are dispersed in a printable conductive ink. One of the main advantages of using this Ag NW ink dispersion is the fact that a very low amount of Ag nanowires can allow the formation of highly conductive networks with very high transparency. For instance, when used as 2-dimensional networks for making transparent electrodes, only 30–50 mg of nanowires are needed per square-meter to reach very low resistivity (less than 100 ohm/sq) at high transparency (more than 90% in the visible spectrum). Contrary to previous works involving sacrificial layers and multi-step printing processes [20], the reported results have been achieved using one single nanosecond pulse and the transferred pixels contain only the Ag NW network and the non-volatile pristine ink additives. A microlens based beam homogenizer combined with micromechanical masks has been employed for obtaining flat top beam profiles and producing the desired pixel shape. In particular, the reported approach enables the laser transfer of Ag NW pixels of a plurality of different shapes (e.g., circular, square or rectangular) and the simultaneous curing of the Ag NW network, owing to the absorption of the laser pulse energy. No sacrificial layer is involved and the transfer is accomplished by exploiting the heating effect of the laser pulse on the Ag NW network and the heat transfer to the PolyethyleneNaphthalate (PEN) substrate, which is a thermoplastic polymer with a glass transition temperature (Tg) at 121 °C. Moreover, we present numerical modelling results of the curing effect of the laser pulses utilized for the printing of Ag NW at the solid state, which sheds light on the feasibility of the single step printing and curing process of Ag NW pixels on PEN substrates. These results indicate that the laser induced heating leads to temperatures above the minimal curing temperature of the Ag NW network and results in increased conductivity with respect to non-irradiated samples. On top of that, the laser energy absorbed is transformed into heat which is conducted by the Ag NW to the PEN substrate, inducing local melting on the polymer. Ag NWs are therefore attached to the polymer making the transfer feasible in contact mode. This localized melting enhances the adhesion of Ag NW on the receiving substrate, circumventing a major issue of processing metal NW networks, which is poor adhesion on the substrate [30].

## 2. Materials and Methods

The Ag NWs ink was commercially provided by Novarials Corporation (Woburn, MA, USA) and has 1% w.t. Ag, in water, isopropanol, polyester resin, ester and glycol. The curing protocol according to the manufacturer, for low Tg substrates, consists of baking at 110 °C for 30 min leading to a mean sheet resistance of <100 Ohm/sq. Quartz donor substrates and PEN Teonex^®^ (Teijin Limited, Tokyo, Japan) 125 μm thick films, were also commercially available products. The PEN glass transition temperature is 121 °C and the melting point is at 269 °C. The Ag Nanoparticle ink dispersion (20% Ag content) used for the fabrication of contact pads for the electrical characterization of the flexible transparent electrodes was purchased by Sun Chemical Corp (Sun Chemical Corporation, Parsippany, NJ, USA). The single step laser transfer and curing process relies on the Laser Induced Forward Transfer Method implemented by the laser experimental set-up described in Reference [26], with the addition of an in-house solid phase printing cell with the capability of vacuum contact [28]. A top hat laser beam profile was achieved using a microlens refractive beam homogenizer provided by SUSS MicroOptics SA (Hauterive, Switzerland). An Nd:YAG nanosecond pulsed laser (pulse width 10 ns) at 532 nm, was used as the laser source, which provided short pulses that were imaged at the interface of quartz donors coated with Ag NW network layers. 100 μL of Ag NW ink was spin coated at 1000 rpm for 1min to create a uniform layer. After coating, the samples were left to dry for 60 min at ambient laboratory conditions. The resulting sheet resistance was measured to be 106 Ohm/sq.

The total light transparency of the Ag NW networks was extracted by measuring the transmittance of the coated layers on PEN substrates at the 400–800 nm part of the spectrum, using the V-770 Jasco UV-Vis spectrometer (JASCO, Easton, MD, USA).

For the electrical characterization of the transferred pixels, a four point probe IV station implementing the Van Der Pauw method was utilized.

**Figure 1:**
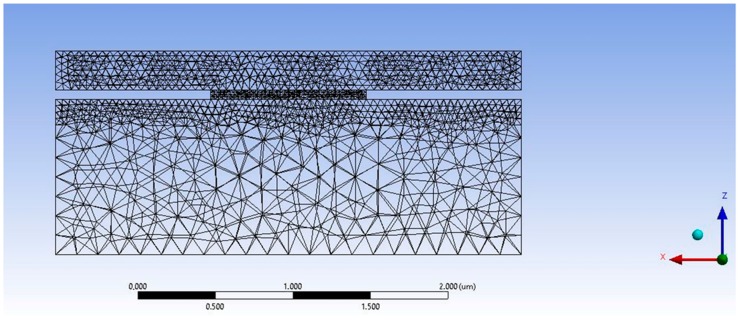
Meshing of the quartz/Ag NW/PEN (top/middle/bottom) interface according to the selected grid for two different nanowire geometries: cylindrical rods and cuboid parallelepipeds.

The commercial software of ANSYS Mechanical (ANSYS Inc, Canonsburg, PA, USA) has been chosen for simulating the lateral and the in-depth temperature profile of the quartz/Ag NW/ PEN interface by conducting a Finite Element Analysis. In Figure 1 the selected Grid is displayed. Grid sensitivity analysis was performed in order to determine the optimal grid in terms of accuracy and time efficiency for our case study: A coarse grid would produce inaccurate results, while a much denser grid approach would be computationally intensive. In our case, a well sized, refined and computationally efficient grid was created, with an average element size of 0.01 nm in the Ag-NW rod body of 50 nm in diameter (total number of elements comprising the rod is 3485). Therefore the aspect ratio of Ag NW diameter:element size is 5000:1. The mesh characteristics of the model are presented in Table 1: The Orthogonal Average Quality is an important measure of the mesh quality and varies from 0 to 1, with the latter being the best possible quality. In our case this value is approximately 0.87, indicating an adequate mesh quality for avoiding discretization errors. Two different geometries were simulated: 1: cylindrical—rod nanowires, 2: cuboid shaped nanowires.

The following table contains the mesh characteristics defined for this model.

## 3. Results and Discussion

### 3.1. Experimental Process

For the purposes of the reported work, a novel process for the laser transfer of solid pixels (comprising Ag NWs), exploiting thermal effects of laser irradiated pixels, has been developed. The process concept relies on Laser Induced Forward Transfer, as reported in previous articles published by the authors [27,28] or other groups [31,32,33,34,35,36], but has several key modifications which enable the intact transfer of solid pixels with well-defined geometrical shapes solely on polymeric and optically transparent substrates. The experimental configuration of the current process involves Laser Transfer from a solid donor layer comprising Ag NWs networks coated in ink form on a donor substrate, and a polymeric PEN receiving substrate with high optical transparency.

**Figure 2:**
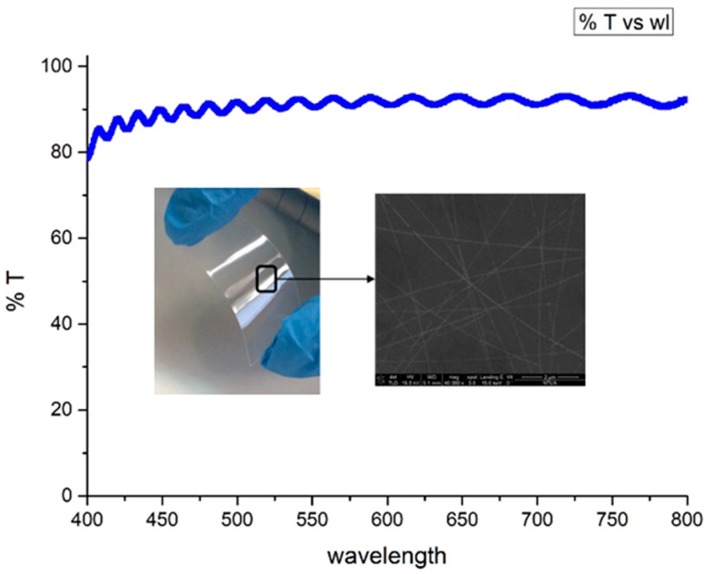
Transmittance vs wavelength for 106 Ohm/sq Ag NW network. Total visible light transmission is over 90% on PEN substrate and T at 532 nm is 91.6% on PEN.

The wavelength of 532 nm was selected because it allows for sufficient absorbance of the laser irradiation by the Ag NW network without inducing plasmonic resonance effects, which would lead to potentially detrimental high temperatures, and at the same time is not absorbed by the quartz and PEN substrates. After coating, the samples were left to dry for 60 min at ambient laboratory conditions. The total visible light transmission was over 90%, as was the transmission for the selected wavelength (see Figure 2). UV-Vis spectroscopy measurements have been conducted to pristine and oven cured spin coated Ag NW networks with no measurable difference.

**Figure 3:**
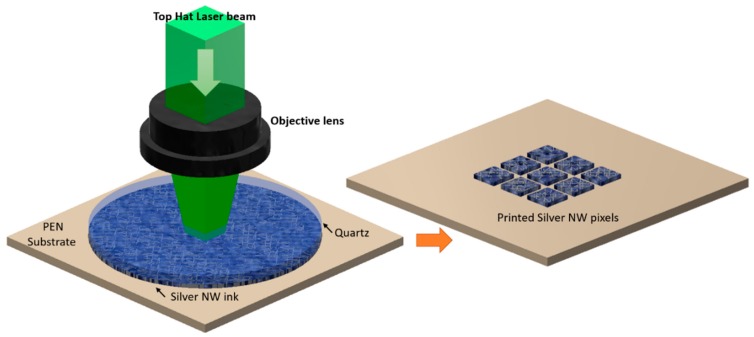
The Single Step Laser Transfer and Laser Curing Process. A top hat laser pulse is imaged at the interface of an Ag NW network coated on the bottom side of a quartz donor layer, donor and receiving substrates being in low vacuum contact.

The Laser Transfer Process mechanism is illustrated in the following figure (Figure 3): A pulse is imaged at the interface of the donor/Ag NW ink layer and a large part of the laser pulse energy is absorbed by the Ag NW network, resulting in the selective heating of the network.

**Figure 4:**
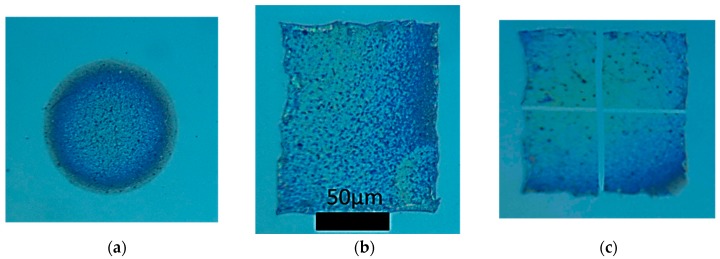
Optical microscope images of solid pixels transferred intact on PEN substrates, using single pulses of 20 mJ/cm^2^ and wavelength of 532 nm. (**a**) circular; (**b**) rectangular; (**c**) Square. An optical filter has been used to enhance contrast.

According to the simulation results and the validation from the electrical characterization presented in Section 3.3 and Section 3.4 respectively, the temperature achieved after a single pulse irradiation is around 230 °C, which is higher than the glass transition temperature of PEN (Tg = 121 °C). The heating effect of the pulse leads to partial melting of PEN within a micrometer sized heat affected zone around each NW, and the subsequent attachment of Ag NWs and the surrounding non-volatile ink additives. With accurate control of laser transfer conditions (e.g., vacuum pressure, laser fluence, beam profile quality), intact pixels can be transferred (see Figure 4). In this figure it is evident that the transferred pixel contains several other additives which sustain the Ag NW in the solution e.g., ester, glycol, and polyester resin. Control over laser fluence in particular is crucial for this specific experiment; as also reported by Araki et al. [20], fluences under 20 mJ/cm^2^ do not allow for efficient transfer of the irradiated pixel, whereas fluences over 40 mJ/cm^2^ have a detrimental thermal effect leading to complete melting of the irradiated Ag NW network. The optimal laser fluence for this experiment was 20–30 mJ/cm^2^. When the PEN substrate is detached from the quartz substrate, delamination or edge-folding may occur, without significant impacts on the transparency or the conductivity of the patterns. Several different shapes of pixels were printed using the imaging of an adjustable micromechanical mask, as is shown in Figure 4. The rectangular shapes in particular provide the opportunity to form long electrodes using single pulses, which have added value for printed electronics.

### 3.2. SEM Characterization

**Figure 5:**
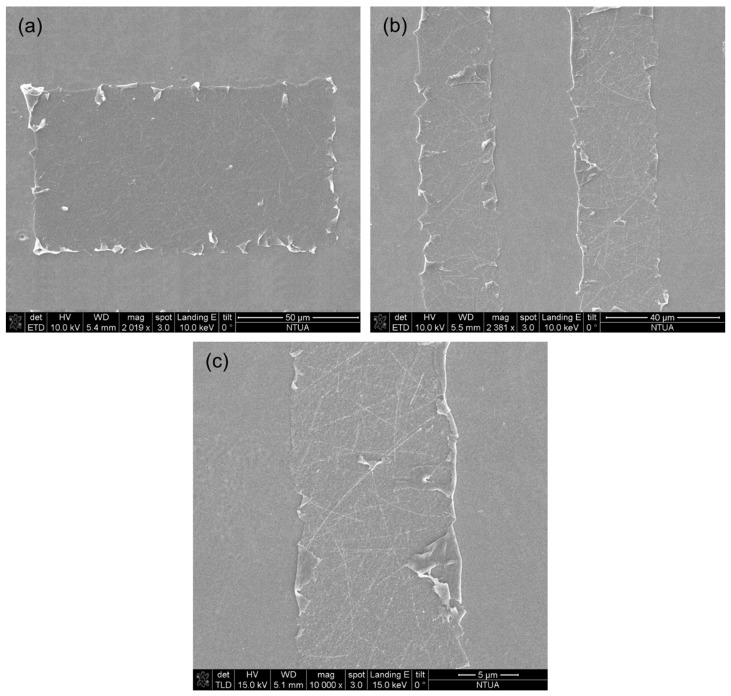
SEM micrographs of printed pixels of high aspect ratio 1:10 rectangular Ag NWs. (**a**) close view of a single pixel. (**b**) two rectangular pixels transferred with a 1:1 ratio of width:spacing. (**c**) Smallest feature size achieved with lateral dimension of 12 μm.

The transferred pixels were thoroughly characterized by Scanning Electron Microscopy. The following figures expose the morphology of the transferred pixels. Even in 2000× magnification, it is evident that the transferred pixels contain a dense network of Ag NWs. With the aforementioned process, rectangular pixels of Ag NW networks sustained by the non-volatile solution’s additives (i.e., glycerol and polyester resin) with an aspect ratio width:length of up to 1:10 and a minimum feature size of 12 μm can be achieved (Figure 5). The transferred pixels suffer from minor delamination issues which do not affect their electrical performance or do not have a significant impact to their optical properties whatsoever. The minimum feature size achieved in this work was in the order of 10 μm (see Figure 5c), but the ultimate resolution of the reported process is only diffraction limited and could go down to around 1 μm after optimization with appropriate configuration of the optics and beam shaper.

**Figure 6:**
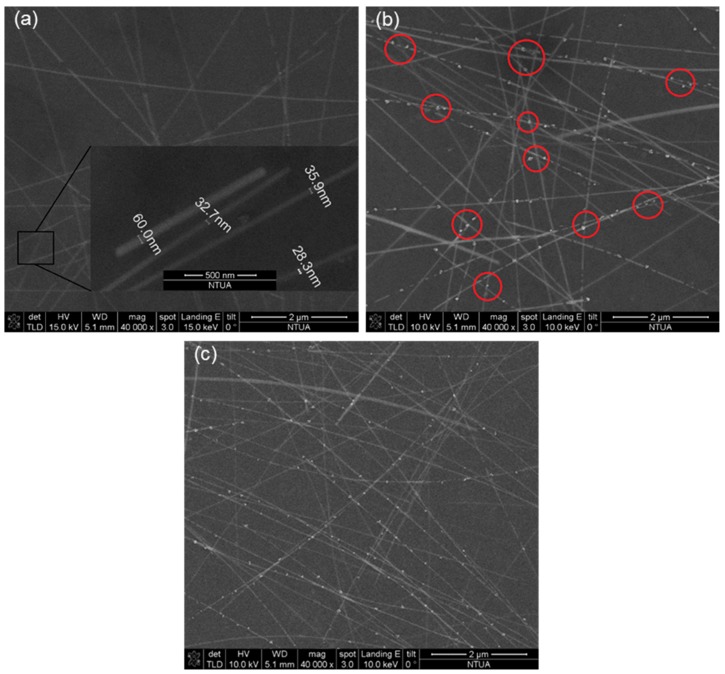
SEM micrographs Ag NW networks: (**a**) Spin coated layer on PEN; (**b**) Laser transferred pixel with curing effect. Fused NW-NW junctions showing balling are marked in red circles. (**c**) Oven cured Ag NW network, with a slightly increased density of fused junctions.

In Figure 6a a micrograph of a pristine Ag NW ink network spin coated on PEN is shown in high magnification. From this figure it is evident that there is a lateral size distribution of the Ag NWs from ~40 to above 80 nm. As thicker NWs will be more massive, they will require higher pulse energies in order to efficiently fuse. Moreover, thicker NWs will have higher thermal conductivities with respect to thinner ones [30], and this will further increase the demand for higher laser fluence for achieving the same temperature. The next Figure 6b, is a high resolution image of the post-transfer, laser cured pixel. The Ag NW network morphology is significantly modified owing to the effect of the laser irradiation. Balling and fusion of Ag NWs is evident across the network. In almost every NW-NW junction, melting has taken place which is exposed as a ball-shaped fused spot. From Figure 6c, which depicts an Ag NW network after oven curing, it is evident that the density of fused NW to NW junctions is slightly increased with respect to Figure 6b. Although this effect could be undesirable for some applications [20], in this specific work we have benefited from the laser induced curing of the Ag NW network, which takes place in parallel with the transfer, to improve the conductivity of the transferred pixels. This topic is discussed in detail in the next section.

### 3.3. Electrical Characterization—Figure of Merit

Ag NW network electrical performance is usually benchmarked against their transparency [37], providing the corresponding figure of merit σ_DC_/σ_OP_, the ratio of the electrical to the optical conductivity.

This ratio can be extracted from the relation of transmittance with the sheet resistance of thin metallic films,

(1) $$T\left(\lambda \right)={\left[1+\frac{188.5}{\mathrm{Rs}}\frac{\sigma_{\mathrm{OP}}\left(\lambda \right)}{\sigma_{\mathrm{DC}}}\right]}^{-2}$$

which has been shown to accurately describe the behavior of Ag NW networks [37].

**Figure 7:**
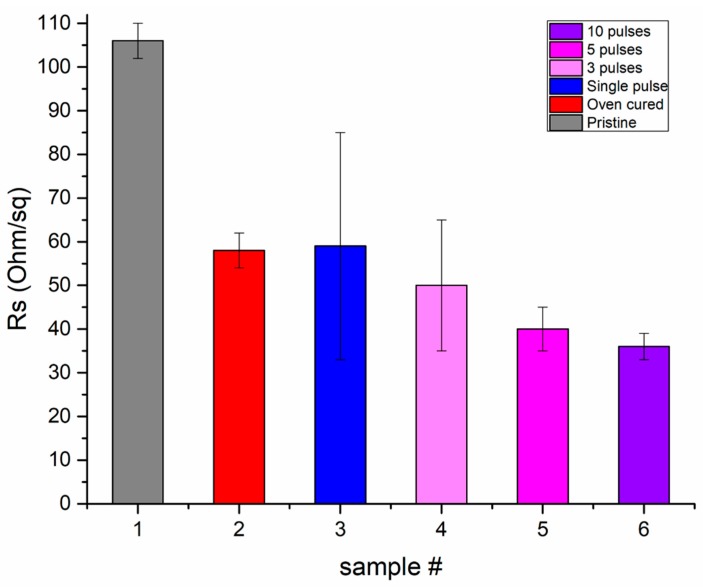
Rs of the pristine Ag NW network layer, after oven baking at 110 °C and post-Laser Curing for 4 different samples: single pulse, 3, 5 and 10 pulse irradiation. In each sample 10 different pixels have been measured. Bars indicate Rs variations from the average value for each case.

In this work, we developed films with fixed total light transmittance 90% and investigated the resulting sheet resistance of the transferred pixels. To achieve that, we designed and implemented a measurement platform by again employing an all laser direct writing process: After the transfer of the desired pixels on the PEN substrates was accomplished, square electrodes consisting of commercially available Ag nanoparticle inks were laser printed and patterned at both edges of each pixel, with at least 10 μm overlap to ensure proper electrical connectivity, and then laser sintered according to the process reported in previous work of the authors [38]. This resulted in fully conductive electrodes with negligible resistance with respect to the Ag NW pixels under investigation. The structures were then measured electrically with a 4—point probe station employing the Van der Pauw method. The resulting sheet resistance values are listed in Table 1. These values are benchmarked against sheet resistance values for layers of the same transparency, but without any laser processing. This comparison is presented in the graph of Figure 7 and listed in Table 2. It is evident that the sheet resistance decrease ranges from 20–70% and this is confirmed by at least 10 different samples. Although this variation range is significant, one can expect a more restricted range of post-LIFT resistance decrease for denser Ag NW networks. Denser Ag NW networks can be formed by either using inks of higher Ag concentration, or by transferring overlapping pixels. However, denser networks have poorer optical transparency and in this work, the single pulse laser transfer of highly transparent pixels is highlighted. The significance of the high optical transparency is reflected by the figure of merit (FoM) achieved. This improvement in the conductivity is important for the application of interest, and the resulting figure of merit (up to 100) is comparable with the typical figures of merit for Ag NW networks achieved by other groups [37]. Furthermore, in order to demonstrate the value of the described process for the reproducible fabrication of reliable conductive electrodes, the following experiment has been conducted: In addition to the single pulse irradiation, pixels have been irradiated with a series of 3, 5 and 10 consecutive pulses. The printed pixels show no evident variation in morphology with respect to single pulse irradiation, whereas the results from the electrical characterization are included in Table 2. Table 2 also contains the calculated figures of merit for 10 different pixels transferred with a single pulse. The Rs values of Ag NWs layers coated on PEN at 1000 rpm and baked at 110 °C for 30 min are also part of this table and Figure 7.

The average Rs value of the single pulse laser transferred pixels (sample #3), with respect to the oven baked samples (sample #2) is very similar. However, the Rs variation between the individual laser transferred pixels is even larger than 100% in some cases, despite the consistency of experimental conditions. This can be explained by taking into consideration the micrometric lateral dimensions of the transferred pixels and the sparse superficial distribution of the Ag NWs: Only a very small percentage of the surface of the pixels is covered by Ag NWs, and the number of NW-NW junctions decreases significantly as the surface area of the pixels decreases. In the case of the pristine or the oven baked layers, measurements were conducted to mm.sq sized areas, and the Rs variation among these measurements is limited to less than 10%. On the contrary, the number of NW-NW becomes significantly smaller for micrometer sized pixels, in which case even as small variation in the number of effectively fused junctions, is reflected as a large variation in Rs. From the same graph, it is obvious that the effect of an increased number of pulses decreases the average Rs, but also significantly decreases the average Rs variation between each measured pixel. In particular, for 3 consecutive pulses (sample #4) Rs variation is ±15 Ohm/sq, for 5 (sample #5) it goes down to ±5 Ohm/sq and finally for 10 pulses (sample #6) all measured pixels show variation in Rs no more than ±3 Ohm/sq. Based on these results one can conclude that by applying consecutive pulses, the Ag NW junctions are more effectively fused, as the total energy transferred by the laser pulses is adequate for achieving fusion on a larger number of NW junctions. Therefore, as the number of pulses increases, the sheet resistance value converges to the minimum Rs value measured for single pulse irradiation, i.e., 33 Ohm/sq and at the same time the Rs variation decreases significantly, so the process may be reproduced and provide very similar results. One can notice that in the case of consecutive laser pulses the average Rs value is even lower than the one achieved by oven curing, but this can be attributed to the special curing for polymeric substrates conducted to the reported samples owing to the temperature restriction imposed by the glass transition temperature of the PEN substrate (121 °C). The electrical characterization study has been conducted before and after adhesion and bend testing: The samples were bent from a fixed radius of curvature of 1 cm to a minimum bending radius of 2 mm, before being relaxed. The process was repeated for 20 cycles. Moreover, all the samples have undergone and survived FrogTape^®^ (ShurTech Brands, LLC, Avon, OH, USA) Delicate Painting Tape adhesion testing, (180° Peel Adhesion to Stainless Steel: 1.65 N/cm from product manufacturer’s datasheet). No measurable change in resistance was observed after the aforementioned tests.

### 3.4. Modelling

Both the transfer and curing mechanisms of the Ag NW pixels under investigation are dominated by thermal effects and the resulting temperatures after the effect of the laser pulse are the topic of the numerical study conducted for the purposes of this paper. In particular, the temperature profile of the proximal in-depth and lateral area surrounding a single Ag NW has been calculated by Finite Element Analysis modelling, in order to shed light on the pixel transfer mechanism. Although the actual experimental process involves the curing of an Ag NW network (with numerous NW-NW junctions), our model simulates the thermal effects induced on a single Ag NW. This can still provide us with reliable results, as the majority of the Ag NWs have very limited junctions and the temperature is expected to rise for each distinct Ag NW independently, according to the proposed model. Furthermore, the energy required for phase changes (e.g., NW-NW fusion during curing) can be considered negligible with respect to the energy involved in heating. Initially, a cylindrical rod Ag NW geometry with a radius of 25 nm and length of 1 μm was designed, so as to have the more realistic simulation. However, as the heat transfer mechanism considered in this problem is purely heat conduction, the interface between a cylinder and a cuboid plane (PEN) is a line, and heat must be conducted via this boundary, which might lead to high temperature gradients. To assess the validity of this geometry, we designed also a cuboid Ag NW shape with the same volume as the rod’s and conducted the same calculations.

For the appropriate scale of the solution, a solver specifically designed for micrometer-scale sized problems (μmKS solver) was employed and room temperature was set at 293 K. The program solves the thermal equation at each node and produces the temperature profile inside the volume for each time step. When a short laser pulse is focused on the surface, it is assumed that the incident photons are absorbed instantaneously to a penetration depth, l_z_, which is inversely proportional to the absorption coefficient α(T).

The amount of energy absorbed is described by the Beer-Lambert’s law. The heating effect of a single laser pulse is well described by the three dimensional heat equation given by (2):

(2) $$Q\left(x,y,z,t\right)=\rho \left(T\right)\mathrm{Cp}\left(T\right)\frac{\partial T\left(x,y,z,t\right)}{\partial t}-\nabla \cdot \left(k\left(T\right)\nabla T\left(x,y,z,t\right)\right)\;$$

The spatial and temporal variation of the temperature and material properties are on the right part of this equation. ρ(Τ) defines the mass density of the material while C_p_ and k are the thermal properties that can be found on Table 3 for PEN, Quartz and Ag NW. The material properties which were fed into the model are the ones provided by the manufacturer in the case of PEN, whereas for the Ag NW, nanoscale dimensions affect the thermal properties and k was introduced according tο Reference [39], in which the thermal conductivity of a single Nanowire with form factor similar to that of the current experiment is investigated and extracted. This thermal conductivity value is decreased by a factor of 2.2 with respect to bulk Ag, which is usual for metal NW networks with small diameters [30], owing to the increased density of extended defects.

In the x-y plane the corresponding spatial heat flow, Q(x,y), is homogeneous because the laser beam profile is top-hat, while in the z-direction, the term Q(z,t) determines the way in which the radiation is absorbed by the material. For materials with homogenous optical properties, this term can be expressed as:

(3) $$Q\left(z,t\right)=I_{\mathrm{Laser}}\left(1-R\right)q\left(t\right)\alpha \left(T\right)e^{-a\left(T\right)\cdot z}$$

I_Laser_ is the energy density of the pulse. R is the reflectivity of the materials at room temperature, while α(Τ) is the absorption coefficient. For the determination of the correct laser energy density input, the actual experimental parameters were fed into the model: Laser fluence 20 mJ/cm^2^ and pulse width 10 ns. All temperature dependencies are considered negligible for this temperature range and are not taken into account in the simulations. The pulse width simulated in this case is 10 ns and relates to q(t), which refers to the temporal dependence of the pulse. In this model the following temporal evolution of the heat flux applied was applied: From 0 ns to 1 ns the heat flux increases linearly in order to achieve the maximal value at 1 ns. From 1 ns to 10 ns the heat input is constant and finally it diminishes linearly up to the 11th ns, when it is again zeroed. e^−a(T)∙z^ refers to the exponential decay of light at normal incidence. The skin depth of bulk Ag at 532 nm (12.35 nm) is comparable with the Ag NW radius (~25 nm). The simulated interface comprises a stack of Quartz/Ag NW/PEN. The presence of quartz has a significant contribution on the maximum temperature that is reached on the Ag-nanowire and on the interface with PEN, as it conducts and dissipates heat a lot more efficiently than PEN (see thermal conductivity, *k*, in Table 3). In the absence of quartz, preliminary results indicated that then the maximum temperature reached at the surface of the nanowire is more than 400 °C.

**Figure 8:**
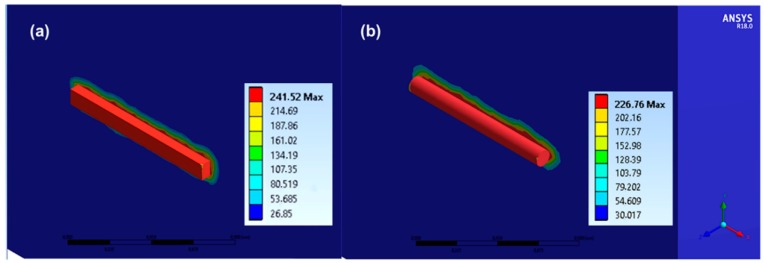
Lateral temperature distribution immediately after the effect of a 10 ns pulse. (**a**) Cuboid parallelepiped geometry, max temperature 241.5 °C. (**b**) Cylindrical geometry, max temperature 226.4 °C. The *x*-*y* plane heat affected zone in both cases is sub-micrometric, and dictated by the geometry of the nanowire.

**Figure 9:**
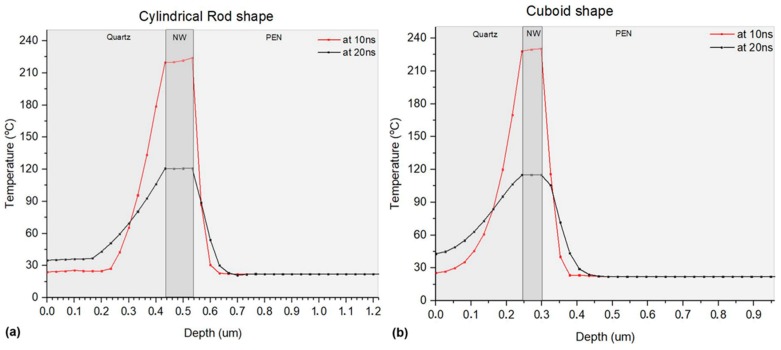
In-depth temperature distribution around the interface of quartz/Ag NW/PEN. Two different time moments are presented: The time corresponding to the maximal achieved temperature, immediately after the end of the effect of the pulse (10 ns), the time of the maximization of the heat affected zone in PEN (20 ns) an (**a**): cylindrical rod shape. At 10 ns the maximal achieved temperature at the interface (226.4 °C) goes down to below 100 °C at 50 nm in the interior of PEN. At 20 ns the heat affected zone is maximized at 100 nm depth. (**b**): cuboid parallelepiped shape. At 10 ns the maximal achieved temperature at the interface (241.5 °C) goes down to below 100 °C at 50 nm in the interior of PEN. At 20 ns the heat affected zone is maximized at 110 nm depth. Heat is also conducted to the interior of the quartz substrate, which is an indication that the presence of quartz contributes significantly to the heat dissipation.

The lateral and the in-depth temperature distributions are presented for both of these geometries in Figure 8 and Figure 9 respectively. These graphs demonstrate the heat distribution after the duration of a 10 ns laser pulse for cuboid and rode shaped Ag-nanowires. The maximal temperature achieved at the surface of the Ag NW is around 230 °C for both geometries. Although the lateral heat affected zone is of the order of the Ag NW dimensions, the in-depth heat affected zone is very restricted. At the Ag NW/ PEN interface the temperature is around 230 °C, and in the depth of 50 nm into the interior of the polymeric substrate the temperature is below 100 °C. Therefore, it is evident that the dimensions of the heat affected zone facilitate the effective attachment of Ag NWs on the surface of the PEN substrate (Tg = 121 °C), without further compromising the body of the substrate (see Figure 9). Temperature rises faster on the quartz side, since its thermal conductivity is significantly higher than the corresponding value of PEN.

The in-depth profile graphs of Figure 9 clearly indicate the high in-depth selectivity of the reported transfer process (heat affected zone restricted to around 100 nm), which relies on heating by short laser pulses (10 ns pulse width). As reported also in previous works [25], nanosecond pulsed lasers are a very efficient and selective tool for the sintering or curing of metal nanostructures. In the current experiment, lateral selectivity is also highlighted, owing to the sparse distribution of the Ag NWs on the x-y plane (see Figure 8). This allows for minimized heating of the PEN surface, localized solely around each Ag NW. The model developed for this specific work could be of generic use for other similar nano-structures and networks, once further verified and validated.

## 4. Conclusions

In this work we have exploited the thermal effect of short laser pulses for the single step laser transfer and laser curing of Ag NW pixels of controllable shape with the lateral resolution down to 10 μm. No sacrificial layer is involved and the transferred pixels consist only of Ag NWs and the non-volatile additives of the ink. LIFT takes place at the solid state with the donor and the receiving substrates being in low vacuum contact. The authors report, relying on both experimental and numerical results, that the driving mechanism which enables the transfer is heat conduction. In particular, the transfer mechanism is considered to be thermal, as the transfer is achieved after a laser pulse is absorbed by Ag NWs which conduct heat on a PEN substrate. Simulation of the temperature distribution at the neighboring area of a single Ag NW has been carried out with the FEA ANSYS mechanical software and the results indicate that the maximal temperature achieved is high enough to selectively melt PEN across a sub-micron sized heat affected zone. The geometry and the dimensions of the Ag NW define the heat affected zone. This gives rise to a very selective heating process with a sub-micrometric heat affected zone, which affects only the neighboring area of each Ag NW and could be of use for a plethora of nanotechnology related applications, e.g., selective heating or annealing of nanocomposite materials or laser processing on a large variety of optically transparent substrates, such as PDMS. This localized melting of the receiving substrate ensures good adhesion with the Ag NW network. At the same time, the simulated temperature value exceeds the curing temperature of the Ag NW network and leads to simultaneous curing of the irradiated areas, resulting in a decrease of the measured sheet resistance by up to 70% for over 90% total visible light transparency. This is translated to figures of merit even higher than 100, which makes the reported process very competitive for the digital fabrication of conductive and optically transparent flexible microelectrodes.

## Figures and Tables

**Table 1:** Mesh characteristics.

Elements	52616
Nodes	89609
Size Function	Adaptive
Conforming Method	Tetrahedrons
Orthogonal Average Quality	0.87

**Table 2:** Electrical measurement for Sheet Resistance and Figure of Merit calculation.

Measurement No	Pristine Rs (Ohm/sq)	Post LIFT Rs (Ohm/sq)	% Rs Relative Decrease	FoM	3 pulses Rs (Ohm/sq)	5 pulses Rs (Ohm/sq)	10 pulses Rs (Ohm/sq)	Oven Cured Rs (Ohm/sq)
1	104	62	40.38	69.7	55	39	37	60
2	106	33	68.87	105.6	45	43	39	58
3	108	53	50.93	82.97	42	44	34	59
4	105	66	37.14	75.76	35	40	33	56
5	105	48	54.28	89.35	39	38	36	58
6	106	85	19.81	41	65	37	35	57
7	108	63	41.66	57.13	63	45	37	59
8	104	34	67.3	102.49	60	36	38	58
9	106	68	35.84	91.7	37	35	35	60
10	108	79	26.85	71.12	61	42	36	56
average	106	59.1	44.306	78.682	50.2	39.9	36	58.1

**Table 3:** Involved Materials’ Properties.

Property	Silver Nanowire	PEN	Quartz
α(T) [cm^−1^]@532nm	8.0996 × 10^5^	negligible	negligible
k [W/mK]	190	0.15	1.3
Cp [J/kgK]	650	1300	800
ρ [gr/cm^3^]	10.49	1.38	2.2
R [-]	0.3	negligible	negligible
